# A Service-Constrained Positioning Strategy for an Autonomous Fleet of Airborne Base Stations

**DOI:** 10.3390/s18103411

**Published:** 2018-10-11

**Authors:** Ferran José-Torra, Antonio Pascual-Iserte, Josep Vidal

**Affiliations:** Department of Signal Theory and Communications, Universitat Politècnica de Catalunya, Barcelona 08034, Spain; fjosetorra@gmail.com (F.J.-T.); josep.vidal@upc.edu (J.V.)

**Keywords:** unmanned aerial vehicle, wireless communications, positioning strategies, navigation, energy consumption, radio resource allocation

## Abstract

This paper proposes a positioning strategy for a fleet of unmanned aerial vehicles (UAVs) airlifting wireless base stations driven by communication constraints. First, two schedulers that model the distribution of resources among users within a single cell are analyzed. Then, an UAV autonomous positioning strategy is developed, based on a fair distribution of the radio resources among all the users of all the cells in a given scenario, in such a way that the user bitrate is the same regardless the users’ distribution and spatial density. Moreover, two realistic constraints are added related to capacity of the backhaul link among the UAVs and the ground station: the bitrate delivered per UAV and the total backhaul bandwidth shared among all the UAVs. Additionally, an energy consumption model is considered to evaluate the efficiency and viability of the proposed strategy. Finally, numerical results in different scenarios are provided to assess both the schedulers performance and the proposed coordinated positioning strategy for the UAVs.

## 1. Introduction

The development of new strategies to enhance the capacity of cellular subscribers has been an important research field for many years. While many systems have already been developed, aerial communications based on unmanned aerial vehicles (UAVs) that are used as airborne base stations (ABBSs), are gaining interest.

UAVs appeared some years ago in tactical applications, mainly to reduce pilot losses in hostile territories. Since then, their cost, size and autonomy have been continuously improved until becoming viable for commercial purposes. Nowadays, many types of UAVs with different capabilities and features are available in the market and allow a huge variety of applications.

In the wireless communication field, UAVs can be used as ABBSs to create non-fixed cellular networks in an ad-hoc way. A new type of network can be designed, in which each base station (BS) is moving continuously depending on the time-varying users spatial distribution and traffic requirements. A possible scenario is, for example, an outdoor sporting event, where a large amount of people is gathered in a small area, whereas the users density remains low outside the venue. Other relevant scenarios for ABBSs are those where the fixed ground network is unreachable (e.g., remote regions in emerging countries) or temporally unavailable (e.g., due to natural disasters). ABBSs have the potential to improve the versatility and availability of wireless services and, as such, they have motivated recent research work.

Many existing works have focused on proposing several air-to-ground channel models [1,2,3,4,5,6,7], which are analyzed in Section 2. More recently, deeper research has been carried out by including different distributions of users in the scenario. According to this, the scenarios can be classified depending on the number of UAVs deployed, as explained next.

Previous works [8,9,10,11] analyze a single UAV scenario. In [8], the authors developed an algorithm that finds the 3-D placement of the UAV that allows serving the maximum number of users. The other three works consider a continuously moving UAV. In [9], beam division multiple access is proposed using millimeter-wave communication and the UAV is moved to combat the blockage problem and improve the user discovery. In [10], three different multiple access strategies are studied, where the UAV moves in response to the users’ activities and positions. Finally, in [11], the moving UAV is used as a mobile cloud to offer computation offloading opportunities to users with limited local processing capabilities. Thus, the UAV is moved to minimize the total mobile energy consumption by also optimizing the bit allocation among users for uplink and downlink communications. In previous works [10,11], several experiments are carried out but without considering the optimization of the altitude of the UAV.

In [12,13,14,15,16], the case of multiple UAVs is considered. Specifically, in [12], an algorithm that aims to serve all users in an specific region with the minimum number of UAVs is developed. The altitudes of UAVs are constant and the signal level received per user is not considered. Thus, the deployment of UAVs is formulated as a geometric problem where a given area has to be served by the minimum number of coverage regions of the same radius with possible overlapping among them. In [13], a scenario consisting of UAVs belonging to different operators is addressed. The authors developed a non-cooperative game theory strategy where rational agents compete to maximize their own individual payoff. Finally, the other three works [14,15,16] focus on heterogeneous networks, where multiple UAVs assist the terrestrial network. In [14], the UAVs are deployed randomly to evaluate their impact on the terrestrial network. In [15], to optimally place the UAVs, the service provided to users is considered. However, the optimal placement is obtained through exhaustive search. In [16], a more sophisticated approach is developed. Firstly, the *k*-means algorithm [17] is used to group the users and, then, taking into account the service, a subset of centroids are selected to be served by a UAV at a given altitude.

In our paper, a novel ABBS autonomous positioning strategy is proposed for the multiple UAVs scenario. As opposed to previous works, the decisions concerning the new 3-D positions of the UAVs are taken under a communication criterion, namely the maximization of the rate delivered to the users subject to fairness and a maximum backhaul bandwidth to be shared among all the UAVs following a frequency division multiplexing (FDM) approach. Some other challenges such as the specific payload and the regulations limiting the altitudes of the ABBSs are deferred to a future work.

The paper is organized as follows. In Section 2, a system overview is given as a background to understand the whole proposed strategy. In Section 3, two different time division multiple access (TDMA) schedulers are evaluated to cover the multiple access challenge for the coverage area corresponding to each individual UAV. Section 4 derives the proposed navigation strategy for the multiple UAVs as an optimization problem where a fair distribution of the resources in the entire scenario is subject to limited rate backhaul constraints that have to be fulfilled. In Section 5, an instantaneous mechanical power consumption model for the UAVs is proposed, and the energy expenditure is estimated. In Section 6, the proposed strategy is evaluated and compared to other works of the state of the art to characterize its behavior. Finally, in Section 7, some conclusions are abstracted and the future work is outlined.

## 2. System Overview

### 2.1. Scenario

We elaborate on a scenario composed of multiple UAVs that have to serve a set of users in a given area. The positions of the users follow a generic spatial density λ(x,y) that may be uniform or not. We assume that the UAVs are initially located at given positions. In the simulations, we consider that the UAVs are distributed uniformly at the beginning, although this is not required. The UAVs have to be able to fly fast to quickly adapt to the static distribution of users but also have to be able to hover (stay fixed in the air) to maintain their positions when the optimum placement of the fleet of UAVs is reached. As reported in [18], there are two main types of UAVs: rotary-blade (quadcopters or hexacopters) and fixed-wing. Thus, according to our requirements, in the sequel, we shall assume the use of rotary-blades UAVs.

Regarding the users-ABBS association, each user is served by a single UAV and a criterion has to be defined to select the most appropriate UAV for each user. The proposed criterion is based on the user-UAV distance $d_{ki}$, where *k* and *i* denote the user and UAV index, respectively. The radiation pattern of the *i*th UAV antenna is denoted by $G_{Ti}$ and is deeply analyzed in the following subsection. Accordingly, the UAV for the *k*th user is selected using the following expression:

(1) $$U\left(k\right)=\arg_{i}\max\left(\frac{G_{Ti}\left(k\right)G_{R}}{d_{ki}^{2}L_{s}}\right),$$

where U(k) is the UAV to be associated to user *k*, $G_{Ti}\left(k\right)$ is the *i* UAV’s antenna gain in the direction of the *k*th user, $G_{R}$ is the gain of the receive antenna, $d_{ki}$ is the distance between the *k*th user and the *i*th UAV, and $L_{s}$ is the loss due to slow fading, that is, shadowing. In the previous expression, we have assumed implicitly that the transmission powers for all ABBSs are equal and constant, which is the most realistic assumption. Note, however, that, if different powers should be incorporated, this could be done by just including the transmission power for each ABBS in the numerator of Equation (1). In this association strategy, we do not include fast fading, as this fading may vary rapidly over time, whereas the association is usually required to be more stable. Note, however, that, if the association could be fast enough to follow the fading changes, then this could be formulated just by including the fading term within expression Equation (1). In the following, and for the sake of simplicity in the notation, we assume that there are no losses due to shadowing (i.e., $L_{s}=1$), although the general case with shadowing could be considered by just incorporating the term $L_{s}$ in Equation (1) and the expressions of the rates that are presented in the following sections.

Note that distances $d_{ki}$ could be obtained through different ways. For example, they could be calculated using global positioning system (GPS) (in a scenario such as this one, it is expected that all the UAVs will be equipped with a GPS unit, and also all user terminals (such as smartphones) will have one, so the GPS coordinates could be used if they are sent to the central unit controlling the positions of the UAVs). Alternatively, the distances among the user devices and the ABBSs could be calculated implicitly using the time-advance mechanisms of the concrete communication standard being considered. In this case, these distances should be reported to the central unit, too. Finally, note that these distances are not strictly needed since each user terminal could estimate the term in Equation (1) for each UAV by just evaluating the equivalent channel gain that encompasses the antenna gains, the fading gain, and the loss due to distance.

This association policy implies a partition of the given area in *P* regions, each one denoted by $A_{i}\;\left(1\leq i\leq P\right)$, where *P* is the number of deployed UAVs and each region contains all the users associated to each UAV. According to this, the received rate per user depends on the capacity delivered by its serving UAV, the number of users in the same region and their positions.

**Figure 1:**
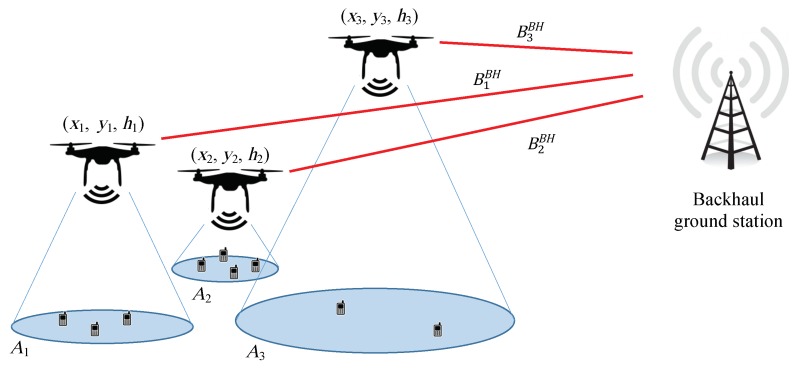
Example of a complete scenario: three ABBSs linked through a wireless connection to a backhaul ground station.

Moreover, the scenario also includes the implementation of an out-of-band wireless backhaul to be shared among all the ABBSs using FDM. Thus, a backhaul link is created for each UAV, which has to support the service delivered to the subscribers attached to each ABBS. The sum of the bandwidths of the individual backhaul links is considered to be limited. In the following sections, it is explained how the proposed iterative algorithm calculates an appropriate distribution of the total backhaul bandwidth among ABBSs to optimize system performance given an initial generic distribution. Again, in the simulations, we consider that this bandwidth is equally distributed among ABBSs al the beginning, although this is not required. Figure 1 represents the scenario.

In Figure 1, the red lines represent the backhaul links between the ABBSs and the backhaul ground station and $\left(x_{i},y_{i},h_{i}\right)$ is the position of the *i*th UAV. As previously mentioned, these coordinates are obtained using the GPS units installed in each UAV and are sent to the central unit controlling the positions of the UAVs. $B_{i}^{BH}$ is the bandwidth of the backhaul link between the *i*th ABBS and the ground station.

### 2.2. Channel Model

Several UAV-user channel models have been proposed and analyzed in the literature. The simplest channel model consists in a direct path between the transmitter and the receiver. It is described by the well known free space path loss [1] through Friis’ equation [19] that depends only on the distance, the frequency and the gains of the antennas. In the ABBS context, it is commonly expressed by the altitude of the UAV and by the elevation angle between the user and the UAV [2,3,4]. Moreover, in [5], a relation between the coverage and the altitude of the UAV is extracted.

In [2,3,4,6,7], more realistic channels models are proposed. Specifically, they include the presence of buildings. This leads to the identification of two different propagation conditions: line-of-sight (LOS) and non-light-of-sight (NLOS). In the first case, a direct communication link between source and destination is possible, whereas, in the latter case, it is not, which leads to additional losses. Each of these cases happens with a given probability of occurrence. In some of these works, the authors also obtained realistic numerical values for the parameters involved in the model. In some of those works, four scenarios are analyzed depending on the distribution of the buildings (suburban, urban, dense urban and high-rise urban) and fading is also taken into account. Finally, another aspect considered in the literature is the movement of the UAVs, which affects the channel coherence time due to the Doppler effect [20].

In this paper, the selected model is the one proposed in [4]. It consists in Friis’ equation plus a fading term due to the presence of buildings in a urban environment. According to this, the received power $P_{r}$ can be expressed as

(2) $$P_{r}=G_{T}G_{R}{\left(\frac{\lambda_{c}}{4\pi d}\right)}^{2}\frac{1}{\xi }P_{t},$$

where $P_{t}$ is the transmission power; $G_{T}$ and $G_{R}$ are the gains of the transmit and receive antennas, respectively; $\lambda_{c}$ is the carrier wavelength; *d* is the transmitter-receiver distance; and ξ is the loss term due to fading in linear scale. Among all of these variables, two require deeper analysis: the antenna gains and the fading.

Regarding the antenna gains, and for the sake of simplicity of the analysis, the user terminal antenna has been selected as isotropic, although the proposed strategy could be extended easily to any other radiation pattern. According to this assumption, the user antenna gain is constant regardless the relative position of the mobile terminal and the ABBS. However, for the UAV antenna, and to obtain more realistic results, the following antennas have been evaluated: omnidirectional in azimuth, half-wave dipole [21,22], Yagi–Uda [21], patch [21] and, finally, a specific antenna used by 3GPP to simulate macro-cell BSs (annex A.2.1.1.1 of [23]). All these antennas lead to a very similar average rate per user when applied in the ABBS scenario. Therefore, the omnidirectional antenna in azimuth has been selected for the UAV, as it presents the simplest gain expression. Its radiation pattern is:

(3) $$G_{T}\left(\theta \right)=\sin^{2}\left(\theta \right)=\frac{h_{i}^{2}}{{\left(x-x_{i}\right)}^{2}+{\left(y-y_{i}\right)}^{2}+h_{i}^{2}},$$

where (x,y,0) is the position of the user, $\left(x_{i},y_{i},h_{i}\right)$ is the position of the UAV serving that user (which is denoted by index *i*) and θ is the elevation angle that can be expressed as

(4) $$\theta =\tan^{-1}\left(\frac{h_{i}}{\sqrt{{\left(x-x_{i}\right)}^{2}+{\left(y-y_{i}\right)}^{2}}}\right).$$

In reference to the fading ξ, as it has been said, two fading terms, $\xi_{LOS}$ and $\xi_{NLOS}$, are possible depending on whether we are in LOS or NLOS conditions, respectively. To define a single model, the following probabilities of occurrence are defined:

(5)PLOS(θ)=11+αe−β(θ−α),(6)PNLOS(θ)=1−PLOS,

where α and β are parameters that depend on the scenario, in this case the urban one [4]. As it can be observed in Equation (5), the LOS probability depends on the elevation angle θ. Thus, Equation (4) has to be applied to obtain $P_{LOS}\left(x-x_{i},y-y_{i},h_{i}\right)$ and $P_{NLOS}\left(x-x_{i},y-y_{i},h_{i}\right)$ as a function of the coordinates of the positions of the user and the UAV. The parameters $\xi_{LOS},\xi_{NLOS},\alpha ,\beta$ and, in general, Equations (5) and (6) are scenario dependent (that is, they will be different in urban, suburban, rural, etc. deployments). Accordingly, it is expected the network operator controlling the positions of the UAVs will have some a-priori knowledge (obtained through drive testing) of those parameters and models.

## 3. Single Cell User Scheduling

The strategy proposed for the positioning of the UAVs aims at a fair distribution of the resources among all the users in the scenario subject to backhaul limitations. As it has been explained in the system overview, the total area is divided in *P* regions, each one served by a single UAV. According to this, a two-step strategy is proposed in this paper. First, a fair distribution of resources among all the users within a single region is performed (which is analyzed in Section 3) and, second, a fair distribution among users in different regions is addressed by taking also into account the backhaul limitations (which is analyzed in Section 4).

To evaluate the distribution of resources among all the users in a given region $A_{i}$ corresponding to the *i*th UAV, two TDMA schedulers have been analyzed. In both cases, the following expression provides the average rate per user $\bar{R}$ in the *i*th region:

(7) $${\left.\bar{R}\right|}_{A_{i}}=\frac{\int \int_{A_{i}}t\left(x,y\right)c\left(x-x_{i},y-y_{i}\right)du}{N_{i}T_{0}},$$

where t(x,y) is the access time assigned by the TDMA scheduler to a user placed at position (x,y) and $c(x-x_{i},y-y_{i})$ is the instantaneous rate per user, which is detailed later in this section. We are assuming that the resource allocation algorithm is able to track the changes due to fast fading. Otherwise, the resource allocation would only be able to adapt to the slow fading and the rate expressions $c(x-x_{i},y-y_{i})$ that are detailed in this section should be replaced by the corresponding ergodic rate. For the single antenna case and for fast Rayleigh fading, the ergodic rate is equal to the rate of the Gaussian channel with a signal to noise ratio (SNR) loss equal to the Euler–Mascheroni constant [24]. In other words, the link SNR budget should consider a fading margin of 2.387 dB.

In the following, du denotes the users differential, $N_{i}$ denotes the number of users to be served in region $A_{i}$ and $T_{0}$ denotes the total time needed to serve all the users in the region, which are expressed as follows:

(8)du=λ(x,y)dA=λ(x,y)dxdy,(9)Ni=∫∫Aiλ(x,y)dxdy,(10)T0=∫∫Ait(x,y)du,

where λ(x,y) is the spatial users’ density, which may be non-uniform, and dA is a differential area. In this paper, we assume a continuous spatial users’ density λ(x,y). This allows obtaining expressions in form of spatial integrals, as is shown below. Note that, if a discrete users’ density is adopted (corresponding to the exact knowledge of all the individual users’ positions and, accordingly, a density function λ(x,y) composed of Dirac delta functions at the users’ positions), then the integrals would instead be expressed as summations. Anyway, all the proposed strategies, results and conclusions would remain the same.

The boundary of the region served by each ABBS can obtained using Equation (1), where it is assumed that each user is associated to a single UAV. In other words, the boundary for region $A_{i}$ is the boundary of the region containing the positions (x,y) for which users are associated to the *i*th UAV. Regarding the instantaneous rate per user, assuming for the moment that there is no fading loss (the impact of fading and LOS/NLOS conditions will be considered in the forthcoming subsections), it can be calculated by plugging Friis’ equation in the Shannon transmission rate:

(11) $$c\left(x-x_{i},y-y_{i}\right)=BW_{A}\log_{2}\left(1+\frac{P_{T}}{\sigma_{n}^{2}}{\left(\frac{\lambda_{c}}{4\pi d}\right)}^{2}G_{T}G_{R}\right),$$

where $BW_{A}$ is the access bandwidth, which is assumed to be the same for all the ABBSs, $P_{T}$ is the transmission power and $\sigma_{n}^{2}$ is the noise power, defined in Equation (12) with $k_{b}$ being Boltzmann’s constant, *T* the temperature and *F* the noise factor in linear scale:

(12) $$\sigma_{n}^{2}=k_{b}\;\cdot \;T\;\cdot \;F\;\cdot \;BW_{A}.$$

Thus, by defining the constant *k* as detailed in Equation (13), $c(x-x_{i},y-y_{i})$ can be rewritten as Equation (14) (where it is assumed that different carrier frequencies are allocated to adjacent UAVs to avoid inter-cell interference):

(13)k=PT·λc216·π2·kb·T·F·GR,(14)c(x−xi,y−yi)=BWAlog21+k·GTBWA·d2.

Two TDMA schedulers are analyzed in the sequel (each one formulated in terms of a different t(x,y) expression), which are the *round robin scheduler* and the *equal rate scheduler*. We assume that the channel is perfectly known, that is, perfect channel state information is available. In practice, the channel should be estimated. Currently, there are several techniques to support the channel estimation process based, for example, on the use of orthogonal training sequences as shown, for example, in [25] for a similar scenario. All these techniques could be used, although we do not consider them since this topic does not lie within the focus of this paper.

### 3.1. Round Robin Scheduler

Round robin consists in the assignment of the same period of time *t* (i.e., t(x,y)=t) to all users in the same region:

(15) $$t\left(x_{k},y_{k}\right)=t\left(x_{j},y_{j}\right)=t,\;\forall k,j,$$

where $t(x_{l},y_{l})$ is the time allocated to a user located at position $\left(x_{l},y_{l}\right)$ in the region. Therefore, Equation (15) leads to the following value of $T_{0}$:

(16) $$T_{0}=\int \int_{A_{i}}t\;\cdot \;du=t\int \int_{A_{i}}\lambda \left(x,y\right)dxdy=t\;\cdot \;N_{i}.$$

Thus, using Equations (7) and (16), the round robin average rate per user is detailed in Equation (17). This rate expression in Equation (17) comes from the the instantaneous rate in Equation (14), where the simplest channel model, assuming no fading loss, has been applied to facilitate the development of the final scheduler expression. However, once the scheduler has been defined, any other channel model proposed in the system overview could be applied. Thus, according to this, the gain of the transmit antenna is changed by Equation (3) and the fading produced by the presence of buildings is introduced, which leads to the more elaborated average rate per user expression in Equation (18), where $\xi_{LOS}$ and $\xi_{NLOS}$ are expressed in linear scale.

(17) $$\begin{array}{cc} {\left.\bar{R}\right|}_{A_{i}} & =\frac{\int \int_{A_{i}}c\left(x-x_{i},y-y_{i}\right)du}{N_{i}^{2}}\\ & =\frac{\int \int_{A_{i}}BW_{A}\;\cdot \;\log_{2}\left(1+\frac{k\;\cdot \;G_{T}}{BW_{A}\;\cdot \;\left({\left(x-x_{i}\right)}^{2}+{\left(y-y_{i}\right)}^{2}+h_{i}^{2}\right)}\right)\lambda \left(x,y\right)dxdy}{{\left(\int \int_{A_{i}}\lambda \left(x,y\right)dxdy\right)}^{2}}, \end{array}$$

(18) $$\begin{array}{cc} {\left.\bar{R}\right|}_{A_{i}}= & \frac{1}{{\left(\int \int_{A_{i}}\lambda \left(x,y\right)dxdy\right)}^{2}}\int \int_{A_{i}}BW_{A}\;\cdot \;(P_{LOS}\left(x-x_{i},y-y_{i},h_{i}\right)\\ & \;\;\cdot \;\log_{2}\left(1+\frac{k\;\cdot \;h_{i}^{2}}{\xi_{LOS}\;\cdot \;BW_{A}\;\cdot \;{\left({\left(x-x_{i}\right)}^{2}+{\left(y-y_{i}\right)}^{2}+h_{i}^{2}\right)}^{2}}\right)\\ & \;\left.+P_{NLOS}\left(x-x_{i},y-y_{i},h_{i}\right)\;\cdot \;\log_{2}\left(1+\frac{k\;\cdot \;h_{i}^{2}}{\xi_{NLOS}\;\cdot \;BW_{A}\;\cdot \;{\left({\left(x-x_{i}\right)}^{2}+{\left(y-y_{i}\right)}^{2}+h_{i}^{2}\right)}^{2}}\right)\right)\lambda \left(x,y\right)dxdy. \end{array}$$

Note that, although the round robin scheduler leads to a simple expression, it has an important disadvantage. All the users are served with the same fraction of time, whereas the instantaneous rate per user depends on the distance between the user and the ABBS, on the radiation pattern of the antenna and on the obstruction of the direct path due to the presence of buildings. Thus, a user placed far away from the UAV will obtain a lower average transmission rate than a closer user, which entails an unfair service among the users in the same region. This is the reason the equal rate scheduler is proposed in the next subsection.

### 3.2. Equal Rate Scheduler

In the equal rate scheduler, the instantaneous rate per user is also considered to assign the fraction of time per user in a way such that all the users obtain the same payload:

(19) $$t\left(x_{k},y_{k}\right)\;\cdot \;c\left(x_{k},y_{k}\right)=t\left(x_{j},y_{j}\right)\;\cdot \;c\left(x_{j},y_{j}\right)=K_{0},\;\forall k,j,$$

where $c(x_{k},y_{k})$ is the instantaneous rate for a user located at position $\left(x_{l},y_{l}\right)$ in the region. Therefore, Equation (19) leads to the following value of $T_{0}$:

(20) $$T_{0}=\int \int_{A_{i}}\frac{K_{0}}{c(x-x_{i},y-y_{i})}\;\cdot \;\lambda \left(x-x_{i},y-y_{i}\right)dxdy.$$

Thus, using Equations (7) and (20), the equal average rate per user is detailed in Equation (21) assuming no fading losses. Regarding the proposed models in the system overview, the same changes as in the round robin scheduler can be applied: the transmit antenna gain is changed by Equation (3) and the fading produced by the presence of buildings is taken into account, which leads to the average rate per user detailed in Equation (22).

(21) $$\begin{array}{c} {\left.\bar{R}\right|}_{A_{i}}=\frac{\int \int_{A_{i}}K_{0}\;\cdot \;du}{N_{i}\;\cdot \;T_{0}}=\frac{1}{\int \int_{A_{i}}\frac{\lambda (x,y)}{BW_{A}\;\cdot \;\log_{2}\left(1+\frac{k\;\cdot \;G_{T}}{BW_{A}\;\cdot \;\left({\left(x-x_{i}\right)}^{2}+{\left(y-y_{i}\right)}^{2}+h_{i}^{2}\right)}\right)}\;dxdy} \end{array}$$

(22) $$\begin{array}{c} \;{\left.\bar{R}\right|}_{A_{i}}\;=\\ \;\frac{1}{\int \int_{A_{i}}\frac{\lambda (x,y)}{BW_{A}}\left(\frac{P_{LOS}\left(x-x_{i},y-y_{i},h_{i}\right)}{\log_{2}\left(1+\frac{k\;\cdot \;h_{i}^{2}}{\xi_{LOS}\;\cdot \;BW_{A}\;\cdot \;{\left({\left(x-x_{i}\right)}^{2}+{\left(y-y_{i}\right)}^{2}+h_{i}^{2}\right)}^{2}}\right)}+\frac{P_{NLOS}\left(x-x_{i},y-y_{i},h_{i}\right)}{\log_{2}\left(1+\frac{k\;\cdot \;h_{i}^{2}}{\xi_{NLOS}\;\cdot \;BW_{A}\;\cdot \;{\left({\left(x-x_{i}\right)}^{2}+{\left(y-y_{i}\right)}^{2}+h_{i}^{2}\right)}^{2}}\right)}\;\right)dxdy} \end{array}$$

Thanks to this strategy, a fair distribution of the resources is fulfilled: the users closer to the ABBS and, therefore, with a higher instantaneous rate, will have a smaller fraction of time and vice versa.

## 4. Positioning and Communication for a Fleet of UAVs

Once the fair distribution of the resources among all the users in the coverage area corresponding to a single UAV has been addressed (see the previous section), we consider now the fair distribution among all users in the multi UAV scenario while taking also into account the restrictions imposed by the backhaul links. To facilitate the notation, in the following, we assume that the intra-cell resource allocation is based on the equal rate scheduler (Section 3.2), although the proposed algorithm could be applied to any other intra-cell scheduling approach.

### 4.1. Problem Formulation

The proposed strategy is based on the maximization of a cost function to achieve a balanced rate distribution among users in different regions subject to several constraints imposed by the backhaul implementation. Specifically, the maximization of the cost function is carried out through the repositioning of the ABBSs and the allocation of bandwidth to the backhaul links. The proposed strategy consists in identifying the region with the lowest average rate per user ($\bar{R}$) and moving the appropriate UAVs in order to increase it. This procedure is repeated until all regions reach the same rate, thus achieving fairness among the service provided by different ABBSs.

Regarding the constraints, the first one consists in limiting the total aggregated rate that each UAV can deliver as, in a real scenario, a BS is limited by the capacity of its backhaul link. The second one consists in limiting the total shared backhaul bandwidth from UAVs to the ground station. This leads to the following optimization problem:

(23)maximize{xi,yi,hi,BiBH}i=1Pmin1≤i≤PR¯Ai(24)subjecttoR¯Ai·Ni≤RiBH,1≤i≤P(25)∑i=1PBiBH≤BBHtotal

where *P* is the number of deployed UAVs, $R_{i}^{BH}$ is the rate supported by the backhaul link corresponding to the *i*th UAV, which is detailed later in Section 4.2.2, $B_{i}^{BH}$ is the bandwidth of this link and $B_{BH}^{total}$ is the total bandwidth that the backhaul ground station supports and that is distributed orthogonally among the UAVs. This implementation of the backhaul is also analyzed in [25] where, moreover, the tracking of each UAV is implemented using beamforming, which could be considered as a future work.

Note that the previous optimization problem in Equation (23) is not convex [26]. The main consequence is that any practical algorithm, such as the one detailed in the following Section 4.2, will only be able to converge to a local optimum, that is, to a globally sub-optimum solution. Anyway, in many situations, such solution will provide good performance. In this paper, this performance is checked by means of simulations in Section 6.

### 4.2. Derivation of the UAV Trajectories

To derive the proposed positioning strategy including the backhaul constraints, an iterative optimization strategy based on [27] is applied. Specifically, at each iteration, the constraints defined in Equations (24) and (25) are evaluated as a first step. If all of them are fulfilled, the cost function defined in Equation (23) is increased by a gradient search algorithm, which leads to an increase of the lowest $\bar{R}$ among all the coverage regions of the UAVs. Otherwise, one of the constraints is randomly chosen among all the unfulfilled constraints [27]. Then, the optimization variables are updated following the gradient of the selected constraint function. This procedure is then applied in an iterative way. Therefore, at each iteration, the implementation is applied through the calculation of the gradient of either the cost function or one of the constraint functions.

#### 4.2.1. Gradient of the Cost Function

Regarding the increase of the cost function, the region with the lowest average rate per user has to be identified and then its rate should be increased, which requires, first, to identify which are the UAVs to be moved and, second, to calculate the optimum movements of these UAVs.

Regarding the first step, the UAVs that have to be moved are those that affect the lowest $\bar{R}$. Observing Equation (22), it is clear that there are two different ways of modifying it. Let us assume that the UAV providing the lowest $\bar{R}$ is $i_{0}$ (i.e., ${\left.\bar{R}\right|}_{A_{i_{0}}}\leq {\left.\bar{R}\right|}_{A_{i}},\;\forall i\neq i_{0}$). Then, the UAVs whose positions affect ${\left.\bar{R}\right|}_{A_{i_{0}}}$ are the $i_{0}$th UAV (that has an impact on the UAV-user distance, the orientation of the antenna, the fading probabilities and the boundary of the region $A_{i_{0}}$) and the neighboring UAVs (that only affect the boundary of the region $A_{i_{0}}$).

As to the second step, the movement of the selected UAVs can be computed through a gradient ascent optimization:

(26) $$v\left[n+1\right]=v\left[n\right]+\mu \;\cdot \;\nabla_{v}\left({\left.\bar{R}\right|}_{A_{i_{0}}}\right)\left[n\right],$$

where μ is the step size, *n* is the iteration index and v is a vector with the positions of all the UAVs and the bandwidths of their individual backhaul links:

(27) $$v={\left[x_{1}\;y_{1}\;h_{1}\;B_{1}^{BH}\;\ldots \;x_{P}\;y_{P}\;h_{P}\;B_{P}^{BH}\right]}^{T},$$

where the superscript *T* stands for transpose.

However, as the gradient has non-zero values only in the entries that belong to the positions of the UAV servicing $A_{i_{0}}$ and its neighbors, the gradients for the other entries of v (that is, the positions of the other UAVs and all the backhaul bandwidths) do not need to be computed.

One of the issues is that a closed form expression of the gradient cannot be found since the boundaries between regions cannot be expressed analytically. Thus, a numerical estimation of the gradient is needed. In this paper, we propose to do it in two stages. First, for each coordinate in vector v corresponding to a $\left(x_{i},y_{i},h_{i}\right)$ triplet for which the movement of the *i*th UAV implies a variation of the cost function, we assume a virtual movement of that UAV in a given direction. Thus, the UAV is not physically moved but a subsequent variation of the average rate per user can be computed using Equation (22) and a UAV test displacement Δ. Then, to estimate the gradient of the *j*th component of vector v, the resulting variation of the average rate per user is divided by Δ:

(28) $$v_{j}\left[n+1\right]=v_{j}\left[n\right]+\mu \;\cdot \;\frac{{\left.\left({\left.\bar{R}\right|}_{A_{i_{0}}}\right)\right|}_{v_{j}\left[n\right]+▵}-{\left.\left({\left.\bar{R}\right|}_{A_{i_{0}}}\right)\right|}_{v_{j}\left[n\right]}}{▵},$$

for all *j* entries with a non-zero gradient.

Moreover, to obtain realistic results, the displacements per iteration (that is, $v_{j}\left[n+1\right]-v_{j}\left[n\right]$ in Equation (28)) are limited by the maximum velocities that a UAV can achieve. These maximum velocities are characteristics obtained from the specifications of the UAV manufacturer and they are the forward ($V_{F}^{Max}$) and the vertical, within positive ($V_{V}^{Max}$) and negative ($V_{V}^{Min}$), velocities. If we denote by $T_{it}$ the time interval associated to each iteration, then the maximum velocity is translated into a maximum displacement constraint given by $V^{Max}\;\cdot \;T_{it}$. In other words, if the displacement calculated by the application of the gradient $v_{j}\left[n+1\right]-v_{j}\left[n\right]$ exceeds this maximum value, then it is limited to the maximum value.

#### 4.2.2. Gradient of the Wireless Backhaul Constraint Functions

As mentioned above, the update of vector v following the gradient of the cost function can only be applied when all the constraints formulated in Equations (24) and (25) are fulfilled. If this does not happen, we need to identify the unfulfilled constraints. A vector w is defined to store the indexes of all the unfulfilled constraints. If w is empty, the cost function is increased according to Section 4.2.1. Otherwise, one of these unfulfilled constraints is randomly selected, which can lead to two different situations: either the selected constraint is one of the *P* constraints defined in Equation (24) or the selected constraint is the one defined in Equation (25). Then, the selected constraint function is used to update the optimization variables through its gradient, as it is detailed below.

Regarding the *P* constraints in Equation (24), each one is related to the individual backhaul link for each UAV whose capacity should be greater than the aggregated rate that is delivered to the users in its service region. To model these wireless backhaul channels, the free space channel model has been used as we assume that the UAVs are positioned sufficiently high and the backhaul ground station is located in an isolated area so that there is no blocking in the backhaul connection (that is, LOS condition). Thus, the backhaul rate that each UAV can use (Equation (29)) depends on its distance to the backhaul ground station $d_{BHi}$ and the backhaul bandwidth $B_{i}^{BH}$ assigned to it. The antennas used for the backhaul are assumed to point towards the ground station. For the sake of simplicity in the notation, we have assumed that both antennas are isotropic and there is no signal occlusion due to buildings:

(29) $$R_{i}^{BH}=B_{i}^{BH}\;\cdot \;\log_{2}\left(1+\frac{k_{BH}}{B_{i}^{BH}\;\cdot \;d_{BHi}^{2}}\right),$$

where $k_{BH}$ has a similar definition to *k* in Equation (13):

(30) $$k_{BH}=\frac{P_{T_{BH}}\;\cdot \;\lambda_{c}^{2}}{16\;\cdot \;\pi^{2}\;\cdot \;k_{b}\;\cdot \;T\;\cdot \;F}\;\cdot \;G_{R}\;\cdot \;G_{T}.$$

Note that the backhaul rate in Equation (29) depends on the distance $d_{BHi}$ such that, if this distance is very large, the corresponding backhaul rate will tend to zero. Since the adaptive positioning strategy being described in this section of the paper looks for the optimum positions of the UAVs, this positioning strategy will prevent the UAVs from going to positions out of the hearing range of the backhaul ground station.

According to the previous expressions, at each iteration, the aggregated rate that each UAV can serve (${\left.\bar{R}\right|}_{A_{i}}\;\cdot \;N_{i}$) is compared to the rate that its backhaul link can carry ($R_{i}^{BH}$), leading to a negative $R_{i}^{BH}-{\left.\bar{R}\right|}_{A_{i}}\;\cdot \;N_{i}$ difference when the individual constraint is not accomplished. Thus, when one of the these *P* constraints is not fulfilled and is randomly selected (namely, that corresponding to the index $i_{p}$), a gradient approach similar to the one defined in Equation (26) is applied to update the optimization variables contained in vector v:

(31) $$v\left[n+1\right]=v\left[n\right]+\mu_{BH}\;\cdot \;\nabla_{v}\left(R_{i_{p}}^{BH}-{\left.\bar{R}\right|}_{A_{i_{p}}}\;\cdot \;N_{i_{p}}\right)\left[n\right],$$

where $i_{p}$ stands for the index corresponding to the selected unfulfilled constraint in Equation (24).

In the computation of the gradient in Equation (31), the entries of v with non-zero values are different from those in Equation (26). These entries can be separated in two groups. On the one hand, those related to the positions of the UAV servicing the region associated to the selected constraint, which affect $R_{i_{p}}^{BH}$, ${\left.\bar{R}\right|}_{A_{i_{p}}}$ and the boundary of $A_{i_{p}}$, and the positions of its neighboring UAVs, which affect the boundary of the selected region $A_{i_{p}}$. On the other hand, the second group of entries in v corresponds only to the backhaul bandwidth of the UAV servicing the selected constraint ($B_{i_{p}}^{BH}$), which only affects $R_{i_{p}}^{BH}$.

As per the gradients of the positions, as it happened with the cost function, a closed form expression cannot be found; thus, a numerical estimation similar to the one formulated in Equation (28) has to be carried out, which is shown in Equation (32), where *j* is the index of entries corresponding to positions with a non-zero gradient:

(32) $$v_{j}\left[n+1\right]=v_{j}\left[n\right]+\mu \;\cdot \;\frac{{\left.\left(R_{i_{p}}^{BH}-{\left.\bar{R}\right|}_{A_{i_{p}}}\;\cdot \;N_{i_{p}}\right)\right|}_{v_{j}\left[n\right]+▵}-{\left.\left(R_{i_{p}}^{BH}-{\left.\bar{R}\right|}_{A_{i_{p}}}\;\cdot \;N_{i_{p}}\right)\right|}_{v_{j}\left[n\right]}}{▵}.$$

Moreover, as it happened in Equation (28), the displacements have to be limited again according to the maximum feasible velocities.

Regarding the case of the backhaul bandwidth, its corresponding gradient can be computed in closed form using the following expression (note that the expression of $R_{i_{p}}^{BH}$ in Equation (29) does not include the region boundaries and ${\left.\bar{R}\right|}_{A_{i_{p}}}$ does not depend on $B_{i_{p}}^{BH}$, thus its derivative is zero):

(33) $$\frac{\partial }{\partial {\left.BW_{BH}\right|}_{A_{i}}}\left({\left.R_{BH}\right|}_{A_{i}}-{\left.\bar{R}\right|}_{A_{i}}\;\cdot \;N_{i}\right)\left[n\right]={\left.\frac{\left(d_{BHi}^{2}\;\cdot \;{\left.BW_{BH}\right|}_{A_{i}}+k_{BH}\right)\;\cdot \;\ln\left(\frac{k_{BH}}{d_{BHi}^{2}\;\cdot \;{\left.BW_{BH}\right|}_{A_{i}}}+1\right)-k_{BH}}{\ln\left(2\right)\;\cdot \;\left(d_{BHi}^{2}\;\cdot \;{\left.BW_{BH}\right|}_{A_{i}}+k_{BH}\right)}\right|}_{n}.$$

In reference to the last constraint in Equation (25), a limitation on the total shared backhaul bandwidth is imposed. When this constraint is not fulfilled and it is randomly selected, a small quantity γ is subtracted from each $B_{i}^{BH}$, as the sum of all the variables $B_{i}^{BH}$ has to be reduced. This reduction has to be small enough so that $B_{i}^{BH}$ is not led to zero. Note that the reduction of $B_{i}^{BH}$ may imply that some of the *P* constraints in Equation (24) become unfulfilled, which should be taken into account in the following iteration of the algorithm.

Finally, to summarize this subsection, Algorithm 1 details the full algorithm with all its steps at each iteration.

**Algorithm 1** Positioning Strategy.1:Start iteration [n]2:**for**  i=1:P **do**3:    **if**
${\left.\bar{R}\right|}_{R_{i}}\;\cdot \;N_{i}\geq R_{i}^{BH}$
**then**4:      Add index *i* to the unfulfilled constraints vector w5:    **end if**6:
**end for**
7:
**if**

$\sum_{j=1}^{P}B_{j}^{BH}\geq B_{BH}^{total}$

**then**
8:    Add the index P+1 to w9:
**end if**
10:**if**w is empty **then**11:    go to 2012:
**else**
13:    Randomly select one constraint of w14:
**end if**
15:**if** selected i≤P
**then**16:    go to 2417:**else** [the selected index is P+1]18:    go to 2719:
**end if**


20:Calculate $\bar{R}$ for each region using Equation (22)21:Identify the region $i_{0}$ with the lowest $\bar{R}$22:Find the UAVs to be moved23:Update the positions of the selected UAVs using Equation (28) and taking into account the restrictions imposed by the maximum feasible velocities. Then, go to 29

24:Find the UAVs to be moved25:Update the positions of the selected UAVs using Equation (32) and taking into account the restrictions imposed by the maximum feasible velocities26:Update $B_{i}^{BH}$ using Equation (33) and go to 28

27:Subtract a portion from all the bandwidths $B_{i}^{BH}$

28:Empty w29:End iteration [n]

### 4.3. Practical Implementation Aspects

Implementation aspects are related to the computational load that each UAV has to support and to the amount of control information exchanged among the UAVs and the ground station. As reported in [18], it depends on the network architecture, which can be decentralized (cellular and ad hoc) or centralized (direct-link and satellite).

The proposed strategy uses a gradient estimation that depends on the position of a certain UAV $i_{1}$, the position of its neighbors and the spatial density of users λ(x,y). To implement this strategy in a decentralized fashion, each UAV has to acquire three pieces of information: (i) the selected UAV $i_{1}$ (it is the UAV corresponding to the lowest rate in case that the constraints are fulfilled, and the UAV associated to the randomly selected constraint in case that any of the constraints in Equation (24) is not fulfilled and is selected); (ii) the positions of all its neighboring UAVs; and (iii) the distribution of the users λ(x,y) or the positions of the user terminals (in that case, integrals of previous equations should be substituted by summations). This distribution can be calculated by each UAV for the users in its serving region as it is expected that each UAV will know the positions of the users assigned to it. Then, this information can be exchanged among UAVs in order to obtain the entire spatial distribution of users. Thus, a lot of information should be continuously sent among all the UAVs, which is the focus in [18], where different routing algorithms are analyzed, that is, different ways of making this information flow among the UAVs in the network. Note that the analysis of the routing strategies is out of the scope of this paper and, therefore, we just assume that this information is available. According to all previous comments, we propose a centralized system to implement this strategy to alleviate the network load at the expenses of less robustness against failures of the central entity.

In a centralized implementation of Algorithm 1, all computation is executed in the backhaul ground station, where the positions of all the UAVs are known and the positions of the users, which are computed by each user terminal, are reported through each UAV to the ground station. Thus, the complete Algorithm 1 is implemented in the backhaul ground station and the new positions of the UAVs are reported back to each UAV. It is important to take into account that this exchange of information spends a small fraction of the backhaul bandwidth $B_{i}^{BH}$ dedicated for each link. This fraction is so small that it almost does not affect the user transmission rate. Note that, if some hovering restrictions apply, due to air safety regulations, they could be easily applied by limiting the value of the gradient associated to the position of the UAV.

## 5. Energy Consumption

Once the positioning strategy has been detailed, in this section, an energy consumption model is introduced. According to [10], in the context where the positioning strategy has been implemented where UAVs are used as ABBSs, two types of energies have to be defined: communication energy ($E_{comm}$) and mechanical energy ($E_{mech}$).

The communication energy is the energy required for a UAV to serve all its associated users and communicate with the ground station through the corresponding backhaul link, and the mechanical energy is associated with the flying mechanisms that keep a UAV in the air performing the maneuvers imposed by the positioning strategy. Each one can be computed as

(34) $$E\left(t\right)=\sum_{\tau <=t}P\left(\tau \right)\;\cdot \;T_{it},$$

where E(t) is the total energy spent until the *t*th iteration, P(τ) is the power consumption at the τth iteration and $T_{it}$ is the time interval associated to each iteration. The battery will be completely spent whenever E(t) exceeds the energy available initially in the battery.

Regarding the communication energy, in the ABBS framework, nano small cells are needed, being particularly characterized by the fact that the consumed power is almost independent of the radiated power, as assessed by the model in [28]. However, the purpose of this paper is the development of a positioning strategy focusing on the movement of the fleet of UAVs. Thus, we neglect in this section the energy consumption associated to communication, assuming that it is supported by dedicated airborne batteries.

Regarding the mechanical energy, an instantaneous mechanical power model ($P_{mech}$) is defined corresponding to the flying maneuvers of a UAV. This power depends on the vertical and forward components of the UAV movement ($V_{V_{i}}$ and $V_{F_{i}}$, respectively):

(35)VFi=(vix[n]−vix[n−1])2+(viy[n]−viy[n−1])2Tit,(36)VVi=viz[n]−viz[n−1]Tit,

where v is the vector detailed in Equation (27), and $i_{x}$, $i_{y}$ and $i_{z}$ denote the *x*, *y* and *z* coordinates of the *i*th UAV.

As stated in Section 2, the required type of UAV is rotary-blade. However, only a few works have studied an instantaneous mechanical power model of this type of UAVs. Some of them only provide practical data extracted from real UAV flights but without an analytical model (e.g., [29]), while others provide models that do not fit our task (e.g., [30]), where the model depends on the instantaneous angular velocity of each propeller instead of the instantaneous velocity of the UAV. Thus, in this paper, we have followed the same methodology as in [31], that is, we adapt the well-known instantaneous mechanical power model of an helicopter ($P_{hel}$) using the flight parameters of a rotary-blade UAV.

This adaptation consists in dividing the weight *W* of the UAV by the number *n* of propellers, as a helicopter has a single propeller, and multiplying the obtained power, which belongs to a single propeller, by *n* to obtain the total instantaneous mechanical power. Moreover, a figure of merit $F_{m}$ is added to model the efficiency of the engines, which typically takes values between 0.7 and 0.8:

(37) $$P_{mech}=\frac{n}{F_{m}}\;\cdot \;P_{hel}\left(W^{\prime }\right),$$

with

(38) $$W^{\prime }=\frac{W}{n}=\frac{(m+M)\;\cdot \;g}{n},$$

where *g* is the gravity constant, *m* is the mass of the UAV and *M* is the load, which in the ABBS context is the mass of the transmission equipment.

The mathematical models for the instantaneous power supplied during the flight have been mainly extracted from [32]. They can be characterized in a different way for the cases of vertical and forward flights.

### 5.1. Vertical Flight

A strictly vertical flight (without horizontal displacements) is divided into three different cases depending on the vertical velocity $V_{V_{i}}$. If $V_{V_{i}}$ is positive, the helicopter is climbing; if $V_{V_{i}}$ is negative, the helicopter is descending; and, if $V_{V_{i}}$ is zero, the helicopter is hovering.

Regarding the climbing and hovering states, the same expression can be used for the supplied power $P_{V}$, which is detailed in Equation (40a), where $V_{Hov}$ is the induced velocity at the rotor in the hovering state defined as

(39) $$V_{Hov}=\sqrt[{}]{\frac{W^{\prime }}{2\;\cdot \;\rho \;\cdot \;\pi \;\cdot \;r^{2}}},$$

where *r* is the propeller blade radius and ρ is the density of air, which depends on the altitude and is detailed later at the end of this section.

In the descending state, the modeling of the power is more difficult as there are different behaviors depending on $V_{V_{i}}$. If $V_{V_{i}}$ is lower than $-2\;\cdot \;V_{Hov}$, we are in the windmill brake state. In this state, the energy is extracted from the air to maintain the rotation, like in a wind turbine, and its consumption is negative which means that the batteries are charged, as detailed in Equation (40c). However, as it is clearly explained in [33], between $-2\;\cdot \;V_{Hov}$ and 0 there are two states that have a more complex behavior. Thus, consequently, they can only be modeled empirically. These states are the vortex ring state (from $-V_{Hov}$ to 0) and the turbulent wake state (from $-2\;\cdot \;V_{Hov}$ to $-V_{Hov}$). They have been studied in different works and some models, which we borrow, have been extracted empirically as in [34]. Specifically, in the vortex ring state, the author of [34] used the same expression as in the climbing state (note that Equation (40a) starts at $-V_{Hov}$ instead of 0) and for the turbulent wake state the expression detailed in Equation (40b) is adopted. Accordingly, Equation (40) summarizes all the possible consumption regimes in vertical maneuvers:

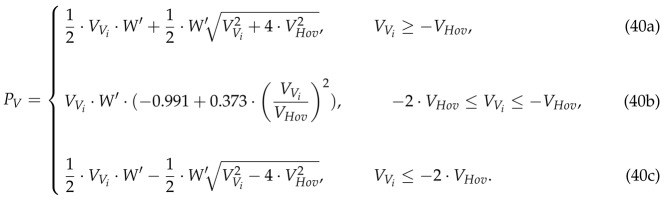


### 5.2. Forward Flight

Regarding the forward flight, it has a single power state that is for positive $V_{F_{i}}$, as neither the UAVs nor the helicopters can fly backward. Moreover, the state $V_{F_{i}}=0$ (hovering) is not contemplated here as it is already done in the vertical flight. As defined in [35], this model has two components, namely the parasitic power and the induced power:

(41) $$P_{F}=P_{par}+P_{ind}.$$

The parasitic power is the power required for overcoming the parasitic drag due to the skin friction of the UAV, form drag, etc. Its expression, extracted from [35], is the following:

(42) $$P_{par}=\frac{1}{2}\;\cdot \;\rho \;\cdot \;C_{D}\;\cdot \;\frac{a}{n}\;\cdot \;V_{F_{i}}^{3},$$

where $C_{D}$ is the drag coefficient, and *a* is the top area of the UAV, which is divided by the number *n* of propellers, as it has been done with the weight.

The induced power is the power required to overcome the lift-induced drag and its expression has been extracted from [32]. It depends on the induced velocity $V_{I}$ at the rotor, whose value in the forward flight has to be found as the solution to the following non-linear equation that does not admit a closed-form solution:

(43) $$V_{I}=\frac{V_{Hov}^{2}}{\sqrt[{}]{{\left(V_{F_{i}}\;\cdot \;\cos\left(\alpha_{P}\right)\right)}^{2}+{\left(V_{F_{i}}\;\cdot \;\sin\left(\alpha_{P}\right)+V_{I}\right)}^{2}}},$$

where $\alpha_{P}$ is the tilt of the UAV, which, for simplicity, is considered constant and always the same in the forward flights. Consequently, a solution has to be calculated numerically. To do it, Filippone [32] introduced the auxiliary variable λ, called induced velocity ratio, which is related with the velocity according to the following expression:

(44) $$\lambda =\frac{V_{F_{i}}\;\cdot \;\sin\left(\alpha_{P}\right)+V_{I}}{\Omega \;\cdot \;r},$$

where Ω is the angular velocity of the propeller, which, for simplicity, has been assumed constant and the same for all the propellers. The numerical value of λ has also to be calculated numerically as the solution to the following equation (which is simpler than the equation formulated in terms of the velocity):

(45) $$\lambda =\mu_{P}\;\cdot \;\tan\left(\alpha_{P}\right)+\frac{\lambda_{H}^{2}}{\sqrt[{}]{\mu_{P}^{2}+\lambda^{2}}},$$

where $\mu_{P}$ is the advance ratio and $\lambda_{H}$ is the induced velocity ratio in hover, which are defined as follows:

(46)μP=VFi·cos(αP)Ω·r,(47)λH=VHovΩ·r,

Therefore, to calculate the numerical value of λ from Equation (45), Filippone [32] proposed to apply the Newton–Raphson method. This iterative procedure is carried out at each iteration of Algorithm 1 to obtain the instantaneous inductive power:

(48) $$P_{ind}=W^{\prime }\;\cdot \;V_{Hov}\;\cdot \;\frac{\lambda }{\lambda_{H}}.$$

Therefore, once the models for the vertical and forward flights have been defined, the final instantaneous supplied power model proposed for the mechanical branch ($P_{mech}$) is the one detailed in Equation (37) with $P_{hel}=P_{V}+P_{F}$, but taking into account two important points. On the one hand, $P_{F}$ is only added for $V_{F_{i}}>0$ to avoid the duplication of the hovering power and, on the other hand, when $V_{F_{i}}>0$ and $V_{V_{i}}=0$, $P_{V}$ has to be neglected as the UAV is doing a forward flight instead of hovering.

There is one last concept that has not been detailed yet, which is the dependence of the model with the altitude of the UAV. As mentioned above, this dependence is modeled by the air density as it changes depending on the altitude [36]:

(49) $$\rho =\frac{M_{m}\;\cdot \;p_{0}\;\cdot \;{\left(1-\frac{L\;\cdot \;h}{T_{0}}\right)}^{\frac{g\;\cdot \;M_{m}}{R\;\cdot \;L}}}{R\;\cdot \;(T_{0}-L\;\cdot \;h)},$$

where $M_{m}$ is the molar mass of dry air (0.0289644 kg/mol), $p_{0}$ is the sea level standard atmospheric pressure (101325 Pa), *L* is the temperature lapse rate (0.0065 K/m), $T_{0}$ is the sea level standard temperature (288.15 K) and *R* is the ideal gas constant (8.31447 J/(mol · K)). Therefore, this dependence is added both in Equations (39) and (42). For simplicity, the altitude is assumed constant during the iteration (the initial one), as $T_{it}$ is low enough to assume that the change of ρ is negligible.

## 6. Evaluation and Results

In this section, some numerical simulations have been carried out to evaluate both the scheduler and the proposed positioning strategy. Table 1, Table 2, and Table 3 presents all the parameters used in the simulations. Some of them have been obtained from the literature and others have been adjusted during the simulations process (μ, $\mu_{BH}$, Δ, γ, $T_{it}$ and grid).

### 6.1. Scheduling Evaluation

The first experiment consists in the evaluation of the two schedulers presented in Section 3 using the models presented in Section 2. The simulations evaluate the aggregated rate obtained by each scheduler in a single cell served by a UAV placed in the center. Thus, in the case of the round robin, this rate is calculated by multiplying the number of users $N_{i}$ in the region by the average rate per user detailed in Equation (18). In the case of the equal rate, $N_{i}$ is multiplied by the user rate detailed in Equation (22).

Although in this experiment only one UAV is considered, the shape of the coverage area has already been selected taking into account the initial distribution of the fleet of UAVs in the proposed strategy, which is implemented in the following experiments. Thus, as this distribution is uniform, the shape of this single region has been selected square. Regarding the distribution of users, a constant users spatial density ($\lambda \left(x,y\right)=\lambda_{0}$) has been considered. It is important to take into account that the value of $\lambda_{0}$ does not modify the plots, as in both schedulers the product ${\left.\bar{R}\right|}_{A_{i}}\;\cdot \;N_{i}$, which is the aggregated rate, leads to the cancellation of $\lambda_{0}$.

**Figure 2:**
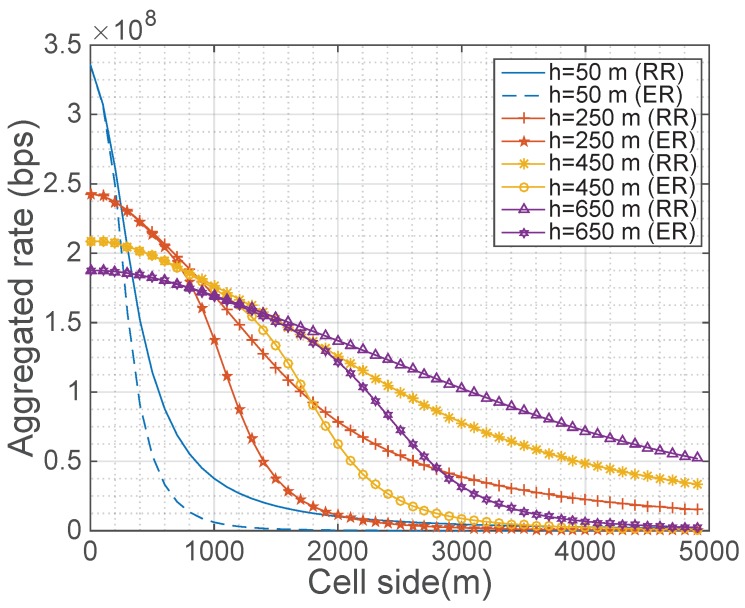
Aggregated rate of the round robin (RR) and the equal rate (ER) schedulers as a function of the cell side.

Figure 2 shows the resulting aggregated rates. As can be checked, the altitude of the UAV impacts differently on the aggregated rate. Specifically, in small regions, the higher the altitude of the UAV is, the lower the aggregated rate is, whereas in big regions the behavior is inverted.

This behavior is related to three different factors, whose dependence with the altitude is also analyzed in [7]: the UAV-user distance, the orientation between the user and the main lobe of the UAV antenna and, finally, the obstruction of the direct channel produced by the presence of buildings. In the case of small regions, all the users are under the main lobe of the UAV antenna and have LOS with the UAV. Thus, the only important factor is the UAV-user distance, which is directly related to the altitude of the UAV. However, in the case of big regions, the users at the cell edge are not under the main lobe of the radiation pattern of the antenna and, consequently, their percentage of NLOS is higher as the elevation angle in the edge is lower. Thus, in big regions, these two factors are more important than the distance and, as a consequence, the dependence of the aggregated rate and the altitude is inverted.

Regarding the effect of this behavior in the positioning strategy, if the regions at the initial step have a side of, for instance, 3000 m, then the altitude of the UAV serving the region with the lowest user rate will increase. However, as the movement of its neighbors tends to reduce the size of the region of this UAV, after some iterations, the region will be small enough and, as a consequence, the altitude of this UAV will start to decrease.

Finally, regarding the comparison between both schedulers, this experiment shows that round robin achieves higher aggregated rates than the equal rate scheduler regardless the size of the region and the UAV altitude. However, this difference is not very high, especially in small regions, and, as mentioned in Section 2, it exhibits a worse user fairness.

### 6.2. Positioning Strategy Evaluation

**Figure 3:**
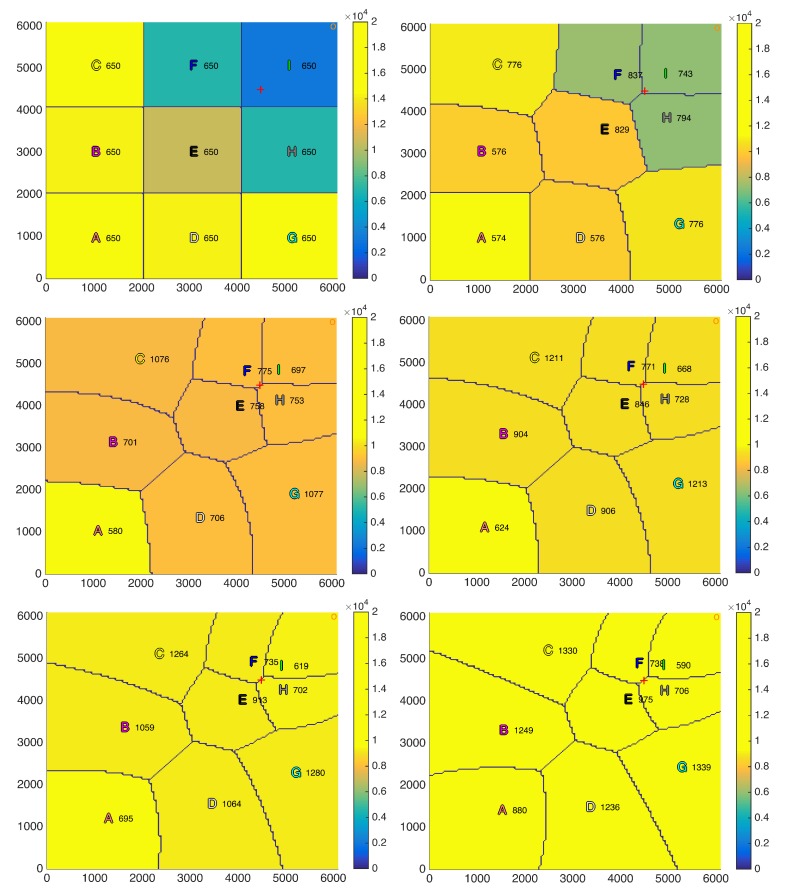
Initial UAV positions and service areas (**top left**); after 500 iterations (**top right**); after 1000 iterations (**center left**); after 1500 iterations (**center right**); after 2000 iterations (**bottom left**); and after 3000 iterations (**bottom right)**.

**Figure 4:**
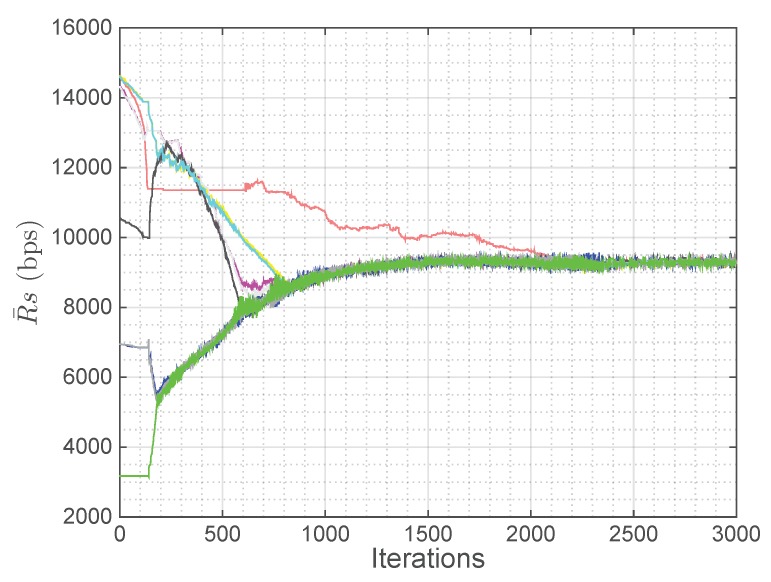
Evolution of the rate per user, for all coverage areas. Each color represents one coverage area using the same colors as the ones assigned to letters in Figure 3.

The positioning strategy derived in Section 4 is evaluated in this subsection assuming that the intra-cell resource allocation is based on the equal rate scheduler described in Section 3.2. A squared scenario with a side of 6100 m and a spatial distribution of users λ(x,y) with a Gaussian spatial shape centered at the point (4500 m, 4500 m) has been considered, which can simulate, for instance, a sporting event scenario. Moreover, nine UAVs have been uniformly distributed in the scenario as starting point, the backhaul ground antenna has been placed at the corner (5900 m, 5900 m) and the total backhaul bandwidth $B_{BH}^{total}$ has been uniformly allocated among all the UAVs as initial distribution ($B_{i}^{BH}=B_{BH}^{total}/P$). Figure 3 shows the scenario in the first iteration and after 500, 1000, 1500, 2000, and 3000 iterations. The peak of the users distribution is plotted as a red cross, the backhaul ground antenna is plotted as an orange circle, the value of the rate for each user at each region $\bar{R}$ is plotted from blue to yellow depending on its value (although it is constant within each region as the equal rate scheduler achieves a fair distribution of the resources) and, finally, UAVs (x,y) coordinates (in meters) are plotted as letters of different colors to be able to track their movements over the iterations. Figure 4 shows the evolution along the iterations of the rate per user ($\bar{R}$) of each region. The colors are the ones defined in the letters in Figure 3.

By observing both figures, it can be seen that, after 3000 iterations, which is equivalent to 15 min ($T_{it}=0.3\;s$), the positioning strategy is able to reduce the difference among the average rate per user of each region by moving almost all the UAVs towards the area with the highest concentration of users (4500 m, 4500 m), which leads to a more uniform distribution of the resources. Figure 3 shows qualitatively how, as the iterations go on, the UAVs move so that the rates for users at different regions tend to be equal. In fact, Figure 3 and Figure 4 show that convergence is almost achieved at iteration 2000.

Moreover, as concluded in the previous experiment, the altitudes of the UAVs serving large regions have increased, whereas the altitudes of the UAVs serving small regions, which are the ones closer to the high density of users area, have been reduced.

Regarding the numerical results of the experiment, we provide two types of results: (1) results evaluating the fairness of the resources distribution among users of different regions; and (2) results evaluating the constraints imposition. In the evaluation of the fairness, we use the Jain’s Fairness index [37], which has the following definition for a vector l of length *L*:

(50) $$f=\frac{{\left(\sum_{i=1}^{L}l\left[i\right]\right)}^{2}}{L\;\cdot \;\sum_{i=1}^{L}{\left(l[i]\right)}^{2}}.$$

This index takes values between zero and one. Zero denotes a totally unfair distribution, whereas one denotes a totally fair distribution corresponding to all the elements in vector l being equal. This index is independent of the scale, the metric and the population size. The index is continuous, which implies that any slight change modifies the fairness index also slightly. To fit it in the multicell scenario, Equation (51) has been derived taking into account that at each region $A_{i}$ there are $N_{i}$ users with the same rate:

(51) $$f=\frac{{\left(\sum_{i=1}^{P}N_{i}\;\cdot \;{\left.\bar{R}\right|}_{A_{i}}\right)}^{2}}{\sum_{i=1}^{P}N_{i}\;\cdot \;\sum_{i=1}^{P}N_{i}\;\cdot \;{\left({\left.\bar{R}\right|}_{A_{i}}\right)}^{2}}.$$

In our simulation, the fairness index has been increased from 0.77006 at the initial step up to 0.99999 in iteration 3000, which was the purpose of the proposed strategy. The rates are 9.29, 9.29, 9.28, 9.28, 9.37, 9.27, 9.27, 9.27, and 9.27 kbps and the average user rate in the entire scenario is 9.29 kbps, which is computed as follows:

(52) $$\bar{R_{T}}=\frac{\sum_{i=1}^{P}{\left.\bar{R}\right|}_{A_{i}}\;\cdot \;N_{i}}{\sum_{i=1}^{P}N_{i}}.$$

These rates are not very high, but we have to consider that the number of users per cell after 3000 iterations is high, around 10,000. Moreover, in real life, not all the users are transmitting simultaneously. Thus, assuming that only 10% of users are transmitting simultaneously, the obtained 10,000 active users per region would correspond to a total number of 100,000 persons in the region, which is a huge population. This amount of users can easily be found in a large sporting facility (there exists some stadiums with a capacity of 100,000 people) placed in a city (the population density of a city such as Barcelona is 15,000 people per km${}^{2}$). In this way, the regions placed outside the stadium still contain a huge concentration of users due to the population density of the city. However, as this density is lower than inside the stadium, these regions are larger.

Note that the proposed equal rate scheduler assures equal rate among the set of active users at a given time. That means that, if either the composition of the group of active users or the traffic profiles change, then the achieved rate may change. A possible solution entails elaborating a more sophisticated resource allocation strategy that forces fairness among all possible sets of active users and traffic profiles. Note, however, that it is very complicated since the scheduler would have to work based on predictions and fairness could only be assured with a certain probability. In fact, fairness should be defined in a different way for each kind of traffic profile. Anyway, the development of this sophisticated scheduler is out of the scope of this paper, whose main objective is to show a framework in which it is proved that performance can be improved by considering jointly resource scheduling and navigation.

Regarding the constraints imposition, although in some iterations there can be unfulfilled constraints, the most important thing is that all of them are fulfilled on average to ensure a proper distribution of the resources. After 3000 iterations the links achieve the following average backhaul capacities $\bar{R_{i}^{BH}}$: 100.01, 111.87, 124.36, 111.77, 143.38, 155.72, 124.57, 155.99, and 173.29 Mbps, which are only slightly higher than their average aggregated rate $\bar{{\left.\bar{R}\right|}_{A_{i}}\;\cdot \;N_{i}}$: 99.31, 106.43, 107.31, 106.28, 139.58, 150.30, 106.99, 151.51, and 135.75 Mbps. The average total shared backhaul bandwidth $\bar{TSBB}$ (179.94 MHz) is lower than total backhaul bandwidth $B_{BH}^{total}$ (180 MHz). Thus, analyzing the results after 3000 iterations, it can be concluded that all the constraints are fulfilled in average, even the constrains in Equation (24), which were not fulfilled in the initialization in the regions far away from the backhaul ground antenna (6100 m, 6100 m).

### 6.3. Comparison to Previous Works

In this subsection, the proposed positioning strategy is compared to different works in the literature where also multiple UAVs are deployed as ABBSs. Specifically, the selected works are by Lyu et al. [12] and Galkin et al. [16] as they are the only ones that fit our evaluated scenario.

To obtain a fair comparison, some adaptations have been carried as it will be explained later in this subsection. It is important to remark that these algorithms do not take into account backhaul constraints considerations. According to this and, in order to evaluate the constraints imposed in the proposed strategy, two stages are carried out. Firstly, the individual bandwidth ($B_{i}^{BH}$) of each backhaul link is computed in such a way that Equation (24) is fulfilled using the minimum required bandwidth. Then, all these bandwidths are added and compared to $B_{BH}^{total}$ to evaluate Equation (25). Therefore, Equation (24) is fulfilled in all the links and the evaluation of Equation (25) defines if the specific strategy accomplishes the total backhaul bandwidth limitation. Finally, in order to compare these algorithms in terms of user rate, the average user rate in the entire scenario is also computed.

#### 6.3.1. Spiral Algorithm

The approach proposed in [12] aims to serve all the users in a specific region with the minimum number of UAVs flying at a fixed altitude. This is formulated as a geometric problem where a given area has to be served by the minimum number of coverage regions with the same radius and with possible overlapping. Moreover, it is developed in a scenario where a number of users are deployed at discrete positions instead of using a continuous spatial distribution of users, which is the case of our evaluated scenario. Thus, the algorithm has been adapted in order to cover the continuous spatial distribution of users in the entire scenario.

**Figure 5:**
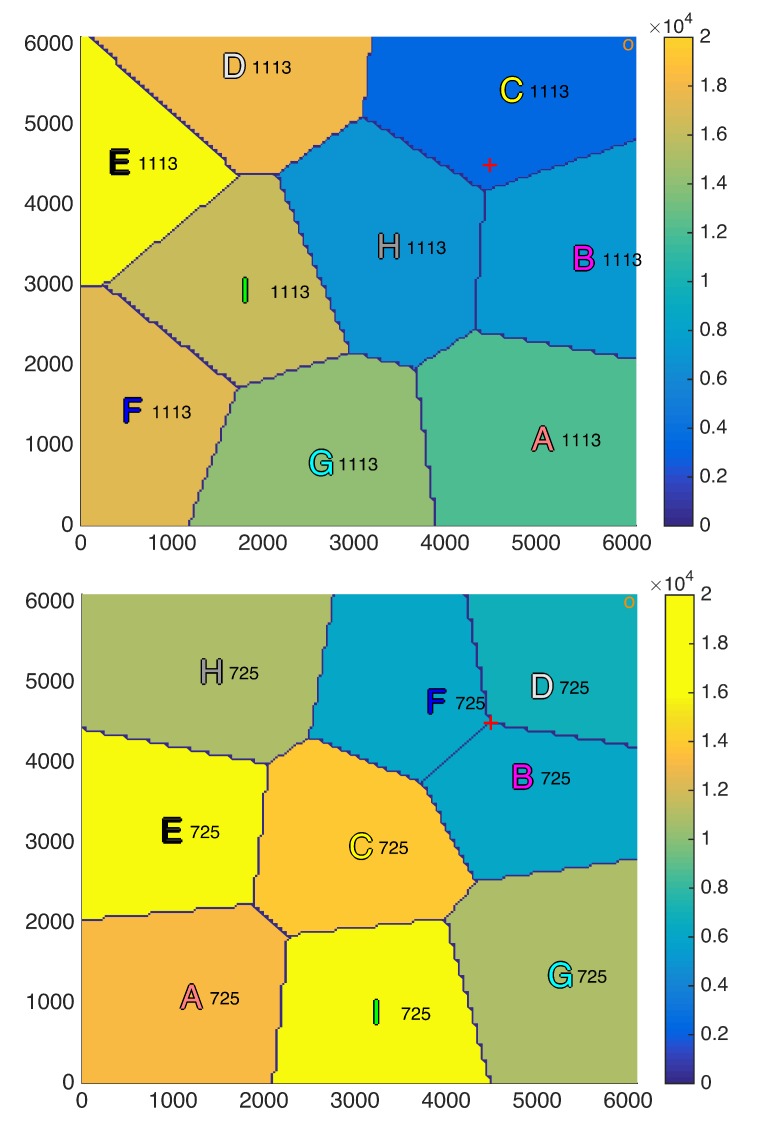
Position of UAVs and coverage areas using: Spiral Algorithm [12] (**top**); and *k*-Means Algorithm [16] (**bottom**).

Figure 5 top shows one possible placement of the UAVs using the spiral algorithm in the scenario described in the previous subsection. However, this algorithm places the first UAV randomly among a set of positions that depend on the border users, which has an important effect on the final placement of all the UAVs. It is important to take it into account as it also implies a variation in the final results of the algorithm. Therefore, although this variability is not important, the results have been averaged over 12 different realizations of the algorithm, which are analyzed in Section 6.3.3.

#### 6.3.2. *k*-Means Algorithm

Regarding the algorithm implemented in [16], it is developed for a scenario where the UAVs assist an already existing terrestrial network. This strategy is defined by two stages, being the first one independent from the terrestrial network. This first stage, which is the *k*-means clustering algorithm, has been used here in order to compare this algorithm with the proposed positioning strategy. Again, this algorithm is developed for a set of users placed at discrete positions instead of a continuous spatial distribution of users. However, in this case, this algorithm cannot be adapted and, as a consequence, a discretization of the spatial distribution λ(x,y) has been carried out.

Figure 5 bottom shows the placement of the UAVs using the *k*-means algorithm in the scenario described previously. In this case, as the convergence of the *k*-means depends on the initialization, different initializations are carried out and the solution with the lowest within-cluster sums of point-to-centroid distances is selected. The results are analyzed in the following subsection.

#### 6.3.3. Comparison

In this subsection, the results obtained with the three algorithms are compared. Table 4 shows these results in terms of three relevant aspects: the average user rate in the entire scenario (Equation (52)), the fairness (Equation (51)) and the total shared backhaul bandwidth (TSBB), which, according to Equation (25), should be lower than $B_{BH}^{total}$ (180 MHz). If it is not fulfilled, the algorithm does not fulfill the constraints imposed by the backhaul implementation, which is marked in gray in the tables.

Observing the results, it can be concluded that the average rate per user is very similar for the three algorithms. Note, however, that the proposed algorithm achieves much better fairness, which means that all users will perceive almost the same quality of service (QoS). This would not be the case for the other two algorithms, where there can be high differences between the rates assigned to the best and the worst users, which is a negative effect to be avoided, if possible. The reason the Spiral and *k*-Means algorithms do not achieve a fairness as high as our proposal is because they locate the UAVs using only geometrical criteria but without taking into account the quantity of traffic generated by the set of users assigned to each single UAV. In addition, the strategy proposed in this paper fulfills the constraints related to the total bandwidth/capacity of the backhaul link. In other words, the proposed solution achieves a higher level of QoS while not exceeding the backhaul capacity.

### 6.4. Non-Static Scenarios

In real life, user terminals can move and the number of UAVs in the fleet can change. Thus, the positioning strategy has to be able to adapt to these changes. In this subsection, the strategy is implemented in three different non-static scenarios to evaluate this adaptation. Specifically, in all of them, the initial scenario is the same as in the previous subsections, but after 2500 iterations, when the algorithm has converged as shown in Figure 4, the scenario starts to change.

#### 6.4.1. One UAV Decays

The first non-static scenario consists in the loss of one UAV, which means that all other UAVs have to cover the temporary non-served users with the highest possible fairness. To evaluate this situation, in this experiment, after 2500 iterations, UAV I (green) is removed.

**Figure 6:**
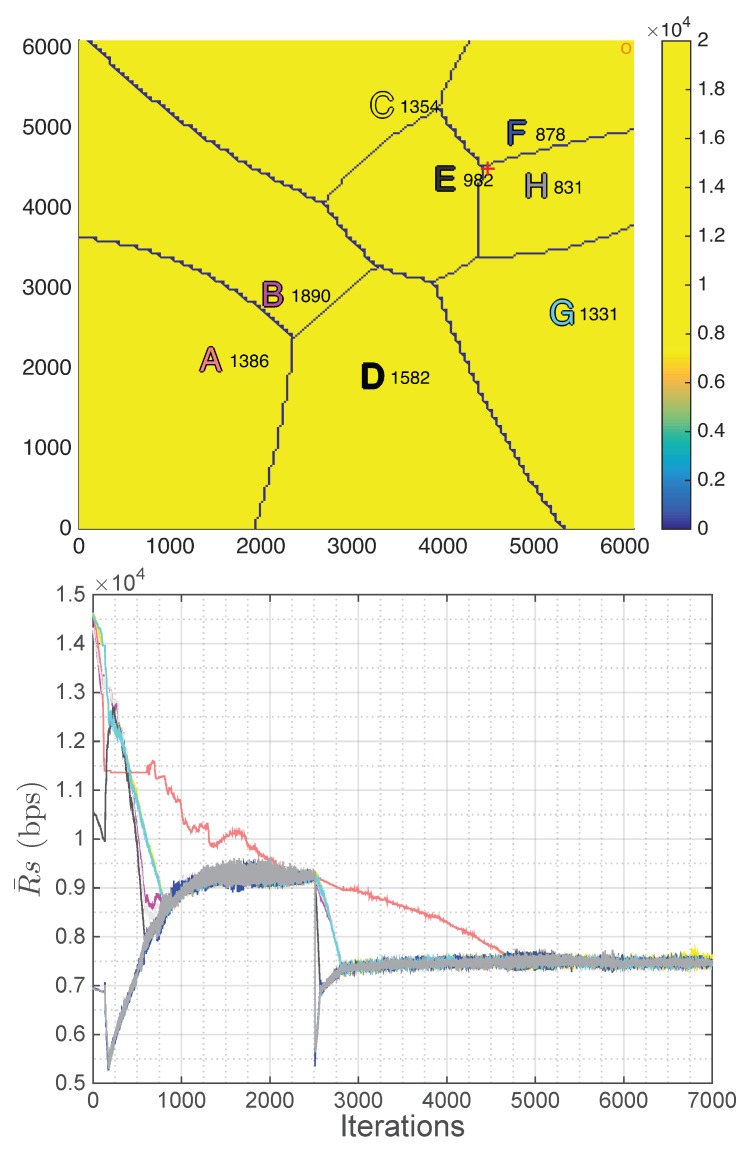
Rearrangement of UAVs when one UAV decays: positions and coverage areas (**top**); and evolution of the rate per user in each coverage area (**bottom**). Each color in the bottom figure represents one coverage area using the same colors as the ones assigned to letters in the top figure.

As can be seen in Figure 6, after 2500 iterations without the decayed UAV, which is equivalent to 12.5 min ($T_{it}=0.3\;s$), the convergence state is reached again. The bitrate per user has been reduced (7.47 kbps), as the same number of users have to be shared among one less UAV, the fairness is maintained very high (0.99999) and all constraints are still fulfilled on average.

#### 6.4.2. Displacement of the Concentration of Users

In the second non-static scenario, after 2500 iterations, the concentration of users, which in the initialization is a Gaussian centered at the point (4500 m, 4500 m), is gradually moved (0.25 m/iteration equivalent to 3 km/h) until the position (1500 m, 4500 m). This scenario could be, for instance, a demonstration, where a big concentration of users is moving simultaneously.

**Figure 7:**
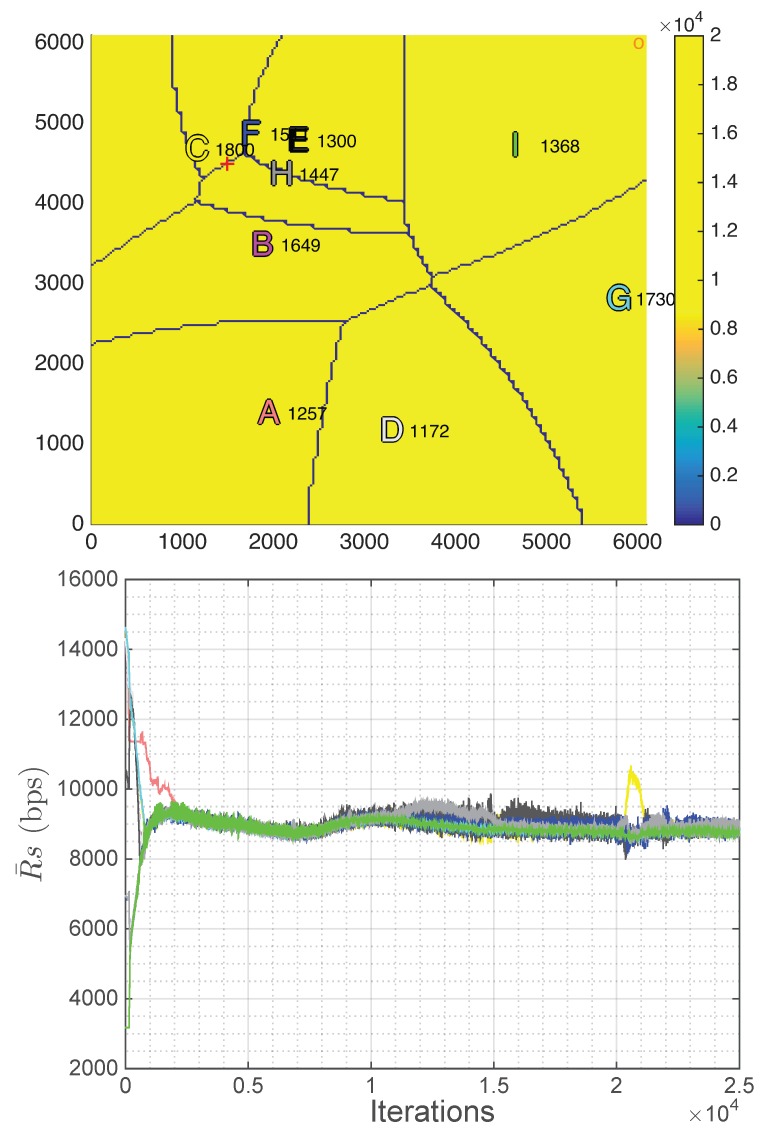
Rearrangement of UAVs when subscribers move: positions and coverage areas (**top**); and evolution of the rate per user in each coverage area (**bottom**). Each color in the bottom figure represents one coverage area using the same colors as the ones assigned to letters in the top figure.

In Figure 7, this displacement of the Gaussian can be deduced by observing the red cross. Moreover, in this figure, some UAVs seem to overlap, but it is only due to the fact that they are at different altitudes and, as a consequence, the upper UAVs serve the far users. Regarding the performance of the proposed strategy, in this case, a more unstable state is reached, in other words, the fluctuation of the rate per user at each region is higher. It is produced by these closer UAVs placed at different altitudes (C, F, E and H), as in this situation a small movement of the UAVs implies a higher variation of the number of associated users. However, this fluctuation is not very high and does not affect the final results of the strategy, as both the achieved fairness and the rate per user are still high (0.99995 and 8.82 kbps, respectively), and all the constraints are still fulfilled on average.

#### 6.4.3. Scattering of the Concentration of Users

In this last non-static scenario, after 2500 iterations, the concentration of users is scattered from a Gaussian to a uniform distribution of users and maintaining the number of users in the scenario. This modification in the distribution of users is carried out by gradually increasing the standard deviation of the Gaussian spatial distribution of the users (0.1 units/iteration) and the number of users in the scenario is controlled with an specific gain that multiplies the Gaussian expression. This scenario could be, for instance, the end of an sporting event, where all the people leave the sporting venue.

**Figure 8:**
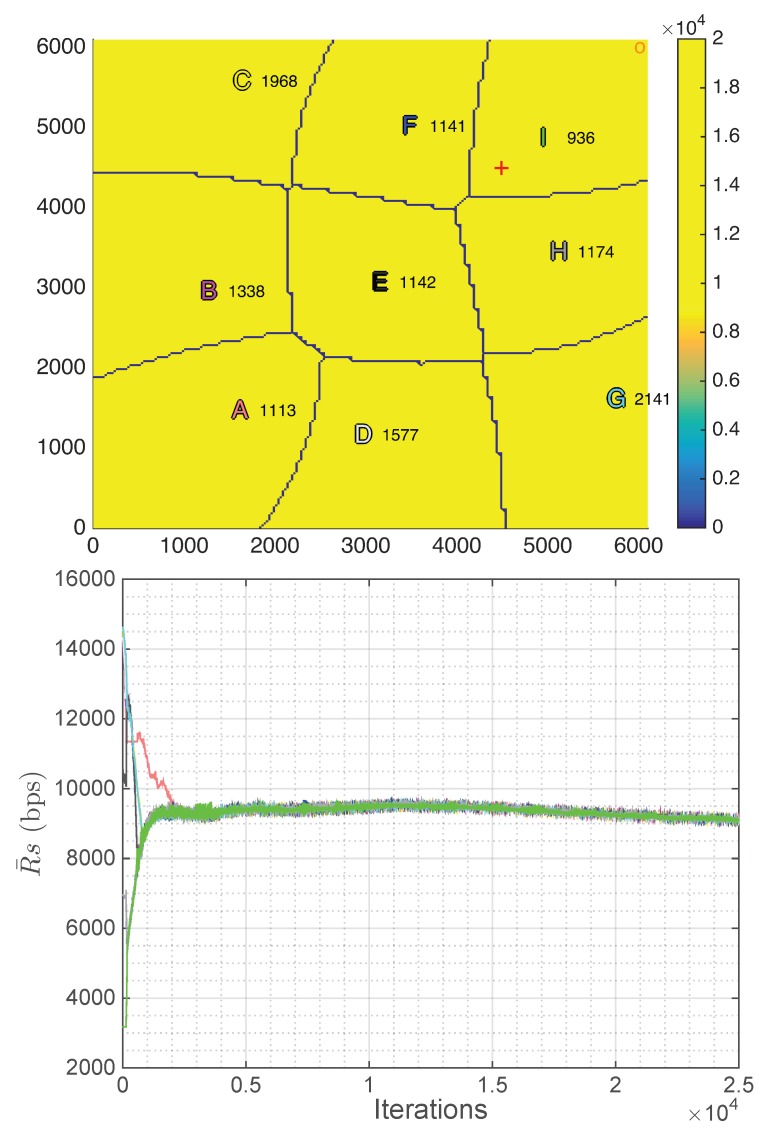
Rearrangement of UAVs when subscribers density returns to uniform: positions and coverage areas (**top**); and evolution of the rate per user in each coverage area (**bottom**). Each color in the bottom figure represents one coverage area using the same colors as the ones assigned to letters in the top figure.

As can be seen in Figure 8, the UAVs tend to distribute uniformly again as in the initial state, which is the expected behavior as the distribution of users tends to also be uniform. Moreover, the results are good, as both the achieved fairness and the rate per user are still high (0.99999 and 9.10 kbps, respectively) and all the constraints are still fulfilled in average.

Therefore, from the observation of the performance of the proposed strategy in the three non-static scenarios, it can be concluded that the proposed strategy is also robust to the changes in the scenario, achieving almost the same results as in the static scenario, which is a very important feature as in real life almost all scenarios undergo changes.

### 6.5. Energy Consumption Evaluation

In this last subsection, the energy consumption in the three previous non-static scenarios is evaluated. As shown in Section 5, some parameters of a real UAV are needed: the number *n* of propellers, the mass *m*, the propeller blade radius *r*, the top area *a* and the maximum velocities. Moreover, it is important to take into account the required type of UAV: rotary-blade. According to this, in this section, a specific UAV has been selected to implement the energy consumption models. It is the hexacopter DJI Matrice 600 (SZ DJI Technology Co., Ltd., Shenzhen, China) [38], whose parameters are listed in Table 2 and Table 3, equipped with six TB48S batteries, which are able to store 129.96 Wh each (equivalent to a total energy of 2807.1 kJ) and a maximum payload capacity of 5.5 kg.

Regarding the communication payload, although the specific transmission equipment has not been analyzed in this paper, nowadays there exist nano Small Cells that only weights a few kilograms. It is the case, for instance, of the IPaccess nanoLTE E40 (IPaccess, Cambridge, UK) [39]. This BS only weights 1.2 kg, supports long term evollution (LTE) cellular communications with a range of 4 km and is able to radiate the power assumed in this paper, i.e., 25 dBm. Therefore, considering that the BS also needs external batteries, another radio for the backhaul link, etc. the total mass of the transmission equipment has been assumed to be 2 kg, which is supported by the selected UAV. This value is an approximation, but the simulations have proved that in the range going from 1.5 to 2.5 kg, which are the expected values of the payload *M*, the variations in the consumptions are very low (±2%).

Once all the parameters of the instantaneous mechanical power model have been detailed, the first experiment consists in evaluating both the vertical and forward models presented in Section 5 for a range of velocities.

**Figure 9:**
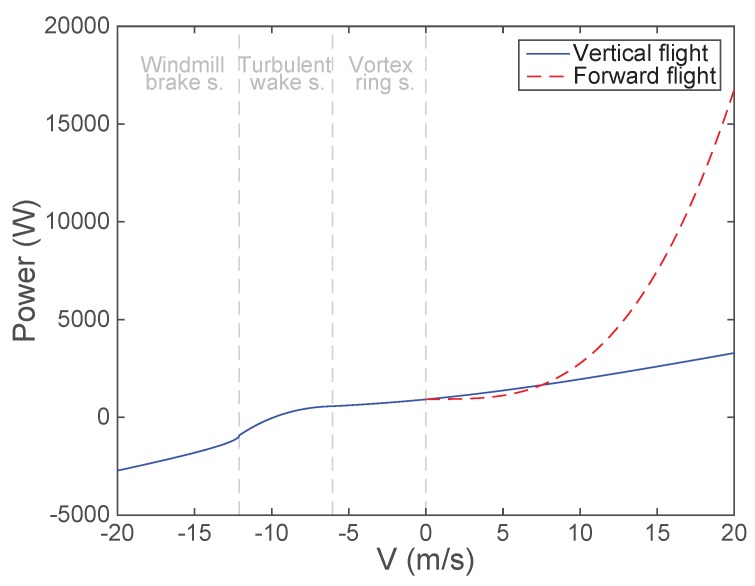
Power consumption models as a function of velocity.

Figure 9 presents both models using an altitude of 600 m to compute the air density in Equation (49). It is important to take into account that, to show real UAV powers, the factor $\frac{n}{F_{m}}$ detailed in Equation (37) has been added in both models. By observing this figure, two important points can be extracted. On the one hand, at V=0, both models lead to the same hovering power, which was the expected behavior and, on the other hand, for $V_{V_{i}}<-1.63\;\cdot \;V_{Hov}$, the obtained power is negative, so the energy is extracted from the air through the reverse movement of propellers, which allows recharging the batteries.

**Figure 10:**
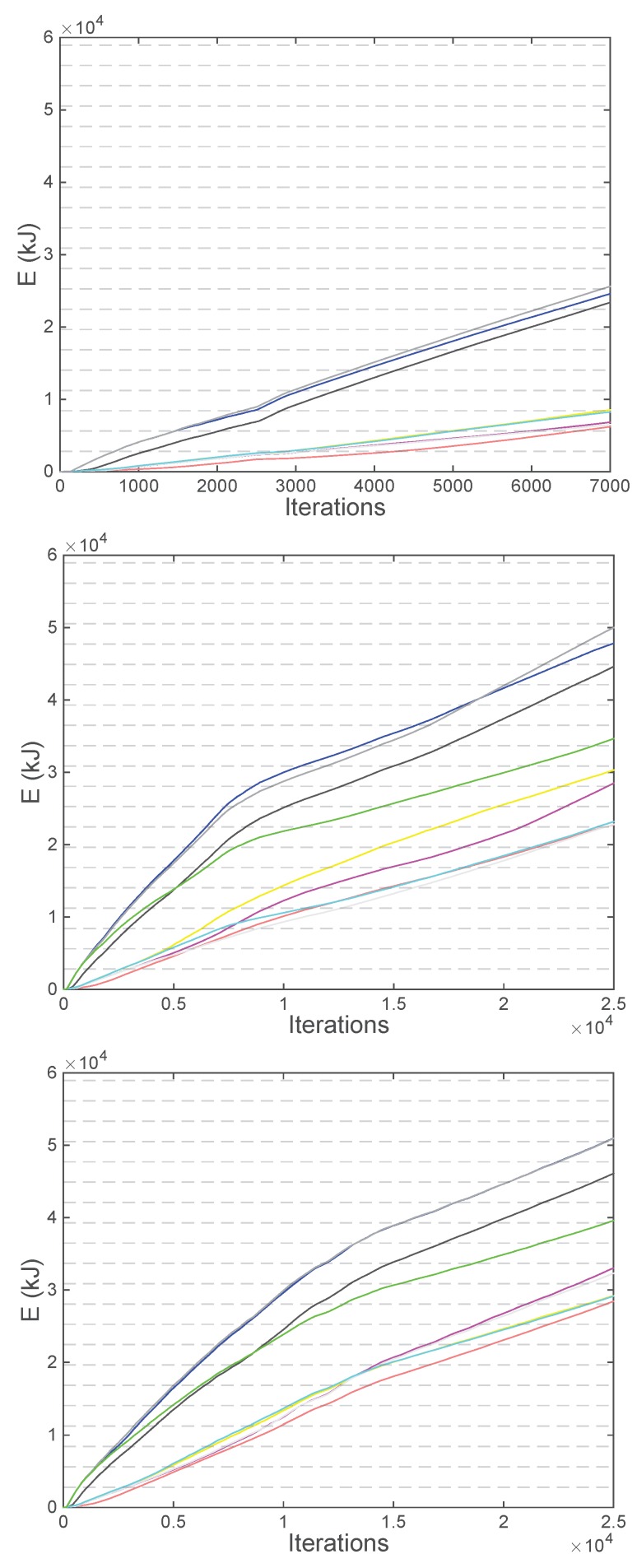
Evolution of the individual energy consumptions vs the numbers of iterations for: one UAV decays (**top**); displacement of users (**center**); and scattering of users (**bottom**). Each color in the figures represents one UAV using the same colors as the ones assigned to letters in the previous figures in this section.

Finally, as the last step of the energy consumption evaluation, the previous models are applied in the three non-static scenarios presented in the previous section. The energy consumed per UAV over the iterations is detailed in Figure 10, where the horizontal dashed lines show the multiples of the energy of the selected UAV (2807.1 kJ).

As can be seen in this figure, each UAV has a different consumption which is related to the maneuvers that have been carried out. Specifically, the highest consumptions belong to the UAVs that have moved fastest in forward flight, which is the expected behavior after analyzing the model shown in Figure 9. In the first non-static scenario, these UAVs are the ones close to the decayed UAV, which are E (black), F (blue) and H (gray), as the new non-served users are assigned to them. Thus, these UAVs need fast displacements to redistribute their big amount of associated users. In the second and the third non-static scenarios, where the users move, the UAVs that have moved fastest in forward flight are the ones close to the peak density of users, which are E (black), F (blue), H (gray) and I (green), as near the peak of users the amount of moving users is higher. Therefore, it implies a higher impact on their associated UAVs and, as a consequence, higher displacements.

Moreover, by comparing the consumptions, two conclusions can be extracted. On the one hand, it can be concluded that the autonomy of the selected UAVs in the evaluated scenarios is very low. Thus, either rotary-blade UAVs with a higher autonomy should be selected, which are difficult to find, or a strategy to replace the UAVs without the loss of rate observed in the first non-static scenario should be developed. On the other hand, taking into account that the iteration time is 0.3 s, it can be seen that the duration of the battery of each UAV is approximately between 5 and 20 min depending on the maneuvers, which agrees with the specifications of the selected UAV [38] taking into account the high altitudes and the payload (2 kg).

## 7. Conclusions

The results show that the proposed strategy can reduce the differences in average rate that each user receives, obtaining a fairness index close to one and fulfilling all the proposed constraints on average, which outperforms considerably the state of the art. Furthermore, the results show that the proposed strategy is robust to changes in the scenario. Finally, regarding the energy consumption, results show that fast forward displacements consumes a lot of energy and that quadcopters and hexacopters with higher autonomies are needed in the ABBS framework.

As a future work, on the one hand, transmission power control is being considered as a way to increase the lowest average rate in addition to UAV movement, where the transmission power would be neither constant over the scenario nor fixed over the iterations. On the other hand, other practical restrictions are being studied, either to obtain more realistic results (e.g., differentiation between active and non-active users, minimum bitrate per user, or upper and lower bounds for flying altitude) or to limit the movement for other benefits (e.g., energy consumption reduction by penalizing the cost function if the obtained displacement requires a high forward speed). A more realistic simulator will also be developed as future work including real data (e.g., a detailed geographical and geometric description of a city, for example, real traffic profiles, etc.).

## Figures and Tables

**Table 1:** General parameters for the communications field.

Parameter	$P_{T}$	$P_{T_{BH}}$	$f_{c}$	$BW_{A}$	$G_{T}$	$G_{R}$	$k_{b}$	*T*	*F*	α
Value	25 dBm	28 dBm	2 GHz	20 MHz	3	3	1.38 $\times 10^{23}$ J/K	290 *K*	5 dB	9.6
**Parameter**	β	$\xi_{LOS}$	$\xi_{NLOS}$	μ	$\mu_{BH}$	Δ	$B_{BH}^{total}$	γ	$T_{it}$	grid
Value	0.28	1 dB	20 dB	3	5 $\times 10^{5}$	52 m	180 MHz	14 kHz	0.3 s	50 m/unit

**Table 2:** General parameters for the power consumption field.

Parameter	$F_{m}$	$C_{D}$	Ω	$\alpha_{P}$	*M*
Value	0.75	1.3	20 rad/s	10${}^{\circ }$	2 Kg

**Table 3:** Specific parameters for the UAV model.

Parameter	*n*	*m*	*r*	*a*	$V_{F}^{\mathrm{Max}}$	$V_{V}^{\mathrm{Max}}$	$V_{V}^{\mathrm{Min}}$
Value	6	9.6 Kg	0.267 m	1.99 m${}^{2}$	18 m/s	5 m/s	−3 m/s

**Table 4:** Results of the different algorithms.

Algorithm	$\bar{R_{T}}$ (kbps)	Fairness	TSBB (MHz)
Proposed	9.29	0.99999	179.94
Spiral	9.40	0.72860	200.64
*k*-Means	9.47	0.86724	187.86
